# Identification of Cohorts with Inflammatory Bowel Disease Amidst Fragmented Clinical Databases via Machine Learning

**DOI:** 10.1007/s10620-025-09323-1

**Published:** 2025-08-13

**Authors:** Matthew Stammers, Stephanie Sartain, J. R. Fraser Cummings, Christopher Kipps, Reza Nouraei, Markus Gwiggner, Cheryl Metcalf, James Batchelor

**Affiliations:** 1 University Hospital Southampton, Tremona Road, Southampton, SO16 6YD UK; 2 Southampton Emerging Therapies and Technologies (SETT) Centre, Southampton, SO16 6YD UK; 3 Clinical Informatics Research Unit (CIRU), Coxford Road, Southampton, SO16 5AF UK; 4 University of Southampton, Southampton, SO17 1BJ UK; 5 ENT Department, Queen’s Medical Centre, Nottingham, NG7 2UH UK; 6 School of Healthcare Enterprise and Innovation, University of Southampton, University of Southampton Science Park, Enterprise Road, Chilworth, Southampton, SO16 7NS UK

**Keywords:** Inflammatory bowel disease, Cohort identification, Data fragmentation, Machine learning

## Abstract

**Purpose:**

Inflammatory bowel disease (IBD) cohort identification typically relies primarily on read/billing codes, which may miss some patients. However, a complete picture cannot typically be obtained due to database fragmentation/missingness. This study used novel cohort retrieval methods to identify the total IBD cohort from a large university teaching hospital with a specialist intestinal failure unit.

**Methods:**

Between 2007 and 2023, 11 clinical databases (ICD10 codes, OPCS4 codes, clinician-entry IBD registry, IBD patient portal, prescriptions, biochemistry, flare line calls, clinic appointments, endoscopy, histopathology, and clinic letters) were identified as having the potential to help identify local patients with IBD. The 11 databases were statistically compared, and a penalized logistic regression (LR) classifier was robustly trained and validated.

**Results:**

The gold-standard validation cohort comprised 2800 patients: 2092(75%) with IBD and 708(25%) without. All the databases contained unique patients that were not covered by the Casemix ICD-10 database. The penalizsed LR model (AUROC:0.85-Validation) confidently identified 8,159 patients with IBD (threshold: 0.496). By combining the likely true-positive predictions from the LR model with likely true-positive IBD clinic letters, a final estimate of *13,048* patients with IBD was obtained. ICD-10 codes combined with medication data identified only 8,048 patients, suggesting that present recapture methods missed *38.3%* of the local cohort.

**Conclusion:**

Diagnostic billing codes and medication data alone cannot accurately identify complete cohorts of individuals with IBD in secondary care. A multimodal cross-database model can partially compensate for this deficit. However, to improve this situation in the future, more robust natural language processing (NLP)-based identification mechanisms will be required*.*

**Supplementary Information:**

The online version contains supplementary material available at 10.1007/s10620-025-09323-1.

## Introduction

- **What is already known on this topic**: *IBD patient cohorts can be identified using billing/read/clinical codes and medication data.*
- **What this study adds**: *Nine additional databases containing unique patients with IBD are identified, and retrieval strategies to overcome database fragmentation demonstrate that medication data and ICD-10 codes only cover 61.7% of the total local cohort.*
- **How this study might affect research, practice, or policy**: *Retrospectively identified patients with IBD are currently missing from population and local-level secondary care cohorts.. IBD prevalence is, therefore, likely systematically underestimated. More advanced cohort identification mechanisms will be required in future.*

Clinical cohort identification challenges vary substantially in difficulty by domain, ranging from comparatively simple conditions like chronic kidney disease (CKD), which is diagnosable purely by the estimated glomerular filtration rate(eGFR) over time [1], to more challenging conditions, such as age-related macular degeneration (AMD) and its subtypes like geographic atrophy (GA), which can take an expert up to half an hour to diagnose visually from a scan, and until recently, had only a single ICD-10 umbrella code (H35.3) [2].

Ulcerative colitis (UC), Crohn’s disease (CD), and inflammatory bowel disease unclassified (IBDU) are chronic inflammatory conditions collectively known as inflammatory bowel disease (IBD) [3]. They are diagnosed using a combination of clinical, biochemical, genetic, radiological, endoscopic, and histopathological tests [4]. The best estimates suggest that the number of patients with IBD is increasing, with over 700,000 patients currently affected in the UK [5]. The cost of care for IBD is demonstrably high, with annual per-patient treatment costs of £3084 and £6146 for UC and CD, respectively [3, 6].

IBD is an interesting test case for a clinical cohort identification study because it is relatively common, with existing national registries and national cohorts [7–10], suggesting a degree of national cohort identification confidence. However, there is no definitive single diagnostic test for the condition, and misdiagnosis may be as high as 10% [11]. Overdiagnosis may lead to medically induced injury, such as overtreatment with medications with significant side effects, and underdiagnosis risks complications directly related to the disease. In one study, 14.7% of patients were lost to follow-up, and 61% subsequently developed a disease flare [12].

Population-level health studies rely on diagnostic billing codes such as ICD-10. In the context of IBD, it has been claimed that diagnostic clinical codes are up to 97% accurate in identifying IBD clinical cohorts [13, 14]. However, this does not fit with real-world experience or other evidence that has consistently shown billing codes to be inaccurate in various clinical contexts [15–20]. In a Danish study conducted in 2020, only 51% of the single-coded CD cohort and 54% of the single-coded UC cohort were accurate [21]. In another study from Scotland, the use of a capture-recapture methodology involving medication data identified 427 previously missed IBD cases [22]. To address this problem, baseline natural language processing (NLP) systems in gastroenterology are at a relatively early stage [23]. This foundational problem must be solved before more advanced NLP systems, such as large language models (LLMs), can be successfully leveraged.

This study highlights the complexities of identifying an IBD clinical cohort using reliable source data collected over 15 years, even within a single institution. It also highlights the risks of data fragmentation and cautions against assuming that prior gold standards, such as ICD-10 codes, are sufficiently robust to be relied upon. Better mechanisms are required to reliably identify patients with IBD (and, by extension, other disease cohorts).

This study aimed to estimate the size of a local IBD cohort across disparate, fragmented databases within a single institution over the past 15 years.

### Objectives

1. Validate a gold-standard IBD cohort.
2. Uncover database patient distributions and usefulness for IBD cohort identification.
3. Explore statistical relationships and comparisons between databases.
4. Estimate the total size of the local IBD cohort using this knowledge.

## Methods

### Inclusion Criteria

All adults aged 18 years or older at the time of their first elective non-two-week wait (2WW) referral to the trust for gastroenterology specialist care between 2007 and 2023, who did not opt out of using their clinical data for research in secondary care, were included in the study. The year 2007 was selected as the start of the study because this was the year the electronic patient administration system (PAS) was installed, and digitized trackable referral data began to accrue.

### Clinical Ethics and Checklist

The Wessex REC and HRA provided research ethics board approval for this study (IC-IBD:23/SC/0152) on 16 May 2023. The study followed the original transparent reporting of a multivariable prediction model for individual prognosis or diagnosis (TRIPOD) checklist [24], as tracked by page numbers in *Supplement 1***.**

### Datasets

Internal databases hosted at the institution were examined and screened to identify a suspected IBD cohort. The 11 separate databases were broadly categorized into four groups.

#### Coded Databases

1. **ICD-10 Diagnosis Codes** (casemix). Registered IBD clinical ICD-10 codes include (‘K50.0,’ ‘K50.1,’ ‘K50.8,’ ‘K50.9,’ ‘K50.X,’ ‘K51.0,’ ‘K51.1,’ ‘K51.2,’ ‘K51.3,’ ‘K51.5,’ ‘K51.8,’ ‘K51.9,’ ‘K51.X,’ ‘K52.3,’ ‘K52.9’) as per the recommended RCP organizational IBD audit [25].
2. **OPCS-4 Surgical Procedure Codes**. OPCS-4 codes that could represent IBD surgery (G58 – G82 & H01 – H56) as per the recommended RCP organizational IBD audit [25]. The definitions of these codes are provided in *Supplement 2*.

#### Registry Databases

3. **Electronic Patient Record (EPR) IBD Registry**—The hospital-integrated clinical support system (HICSS™) IBD Patient Module. In this module, patients can only be registered with a diagnosis by a gastroenterology consultant, a specialist registrar or a fellow.
4. **IBD Patient Portal—**(My Medical Record™): Patients can self-register for the platform but can only be added to the IBD pathway by a clinician.

#### Event Databases

5. **Appointments:** Patient Appointments with Gastroenterology. This filter only flagged patients explicitly seen by an IBD specialist as suspected of having IBD.
6. **Lab Biochemistry:** Fecal calprotectins recorded in the laboratory. Only patients with levels > 50ug/L (the lab upper limit of normal) were suspected to have IBD.
7. **Flare Line:** Recorded calls to the nurse-led flare line. As this line also locally covers coeliac disease and other queries, only those with a recorded diagnosis of IBD on the call template were considered to have suspected IBD.
8. **Cytokine Modulator Prescriptions:** Any patient with a documented prescription for a cytokine modulator under Gastroenterology on the EPR was suspected to have IBD.

#### Free-Text Databases

The screening process for these databases is explained in the Free-Text Normalization & Handling section.

9. Gastroenterology Clinic Letters
10. Endoscopy Records
11. Gastrointestinal Histopathology Records

### Primary and Secondary Outcome

The primary outcome of interest was the estimated number of patients in the IBD cohort.

The secondary outcomes of interest included precision (PPV), recall(sensitivity), and F1 score for each database and model to detect IBD diagnoses correctly against the gold-standard cohort, database cardinality, and algorithm fairness.

### Strongly Supervised Gold-Standard Validation Cohort Derivation

The validation cohort was randomly selected from a larger group of patients within at least two of the 11 validation databases listed above. A strongly supervised randomized validation cohort was selected to maximize robustness.

A team of three junior doctors, led by a gastroenterology registrar (SS), performed manual chart reviews of this randomly selected cohort. Each participant was blinded to the efforts of others. They were supervised by a consultant (MS) who oversaw and re-checked each validation unblinding. In the first iteration, microscopic colitis was included for local service reasons, but following peer review, these patients were removed and the experiment was re-run. All other forms of colitis, including radiation, infective, diverticular, ischemic, and drug-related, were also excluded.

### Validation Sample Size Calculation

This study aimed not only to calculate the total size of the cohort but also to build a model to identify individuals using a logistic regression classifier. Therefore, rather than simply relying on only 20 events per variable [26] (EPV) to calculate the sample size, the sample size estimation method described by Pate and Riley was used [27] because this method has been validated in clinical contexts.

The formula for binary predictions follows the logic explained below (*Eq. *1):

**Equation **1**:** Pate & Riley Binary Prediction Sample Size Estimation Formula

1 $$\mathbf{N} = \frac{K_{1}}{p \cdot (1 - p) \cdot S \cdot \ln\left(1 - R^{2}\right)}$$

Equation 1 explains the sample size calculation formula for binary classification models as developed by Pate&Riley [27] where N is the required sample size, K is the number of candidate predictors, S is the desired shrinkage factor, and R^2^ is the expected Cox-Snell R^2^. Additionally, the formula adjusts for the outcome prevalence (p) as displayed, where K_1_ is the effective sample size (derived from the initial equation without prevalence adjustment), and p is the overall prevalence of the outcome.

A binary classification model with an expected Cox-Snell R-squared value of less than 0.05 was chosen because the discriminative value of each clinical dataset was expected to be low. Up to 11 predictor parameters were fed into the model, corresponding to the 11 databases. Allowable validation shrinkage was set at 0.9 among a target population of gastroenterology referrals, where we already know that at least 16.5% will have IBD [28].

Based on this calculation, the gold-standard validation cohort required to train a model must comprise at least 1730 patients with a corresponding validation cohort of at least 519 and a training cohort of at least 1211. A base cohort of > 50% was derived to ensure sufficient scale and power for the study. The complete Python code for this calculation is provided open source for transparency.

### Free-Text Normalization and Handling

All free-text documents were extracted in native format from the EPR and converted into simple strings. The Unified Medical Language System (UMLS) [29] (MRCONSO meta-thesaurus) was then used to remap IBD synonyms across all free text to create normalized terms for IBD in the following list: [“Ulcerative Colitis,” “Crohn’s Disease,” “IBD” (includes IBD-U), “Inflammatory Bowel Disease,” “Proctitis”].

#### Regex Natural Language Processing (NLP) Model

To flag free-text documents as suggestive of IBD, a simple regex-based NLP model was utilized to match the strings according to the following five regular expressions and associated IBD-related lowercase terms:

- (r‘*olitis’ (ulcerative colitis, pan-colitis, and inflammatory colitis)
- r‘*rohn*’ (crohn’s, crohn’s disease)
- r‘*octitis’ (proctitis)
- r‘*flammatory bowel disease’ (inflammatory bowel disease)
- **r‘ibd’** (ibd, ibd-u, ibdu)

### Statistical Analysis

Missing values were imputed as 0 (not-IBD) to maximize the chances of successfully examining the effects of database gaps on cohort identification in real-world practice. This causes the logistic regression (LR) model to underestimate the total cohort size, but it has the benefit of reducing the false-positive rate.

However, the Jaccard index-based cohort estimation system is unaffected and continues to create an overestimation. This allowed the upper and lower cohort size estimates to be established quickly. Means and medians were calculated as appropriate, depending on the skewness, using 95% confidence intervals (CI) or 25th/75th quantiles, as applicable. The kurtosis was also assessed. The 95% confidence intervals were computed using 1000-fold bootstrapping.

#### Jaccard Similarity Index

The Jaccard similarity index [30] (Eq. 2) was used to compare the overlaps in database content statistically. The overlap is defined as the size of the intersection divided by the size of the union of the two sample sets.

**Equation **2**:** Jaccard Similarity Index Calculation Formula

2 $$J(A, B) = \frac{|A \cap B|}{|A \cup B|}$$

Equation 2 demonstrates the formula for the Jaccard Similarity Index (J) between two sets, nominally (A) and (B) in the formula

Jaccard index thresholding is somewhat subjective and dependent on context and task. However, at a basic level, when comparing databases in this context, a level of > 0.75 would typically be considered high and a level of < 0.35 low [31, 32].

#### Plotting and Statistics

Plotting was performed using Python 3.10.10 with the packages matplotlib [33], seaborn [34], and bokeh [35]. Table 1 lists the performance metrics of interest.

**Table 1:** Performance metrics used in this study

Term	Description
Accuracy	The percentage of results that were correct among all results from the system. Calc: (TP + TN)/(TP + FP + TN + FN)
Precision (PPV)	Also called positive predictive value (PPV), it is the percentage of true-positive results among all results that the system flagged as positive. Calc: TP/(TP + FP)
Negative predictive value (NPV)	The percentage of results that were true negative (TN) among all results that the system flagged as negative. Calc: TN/(TN + FN)
Recall	Also called sensitivity, it is the percentage of results flagged positive among all results that should have been obtained. Calc: TP/(TP + FN)
Specificity	The percentage of results that were flagged negative among all negative results. Calc: TN/(TN + FP)
F1 score	In this case, the harmonic mean of PPV/precision and sensitivity/recall is unweighted. Calc: 2 × (Precision x Recall)/(Precision + Recall)

Performance Metrics used in the study

*TP* true positive, *FP* false positive, *FN* false negative, *TP* true negative

Precision (PPV) was selected as the primary outcome measure to rank the databases because it provides the most helpful measure of database performance for identifying IBD cohorts.

#### Jaccard Index Union-Size Calculation

The Jaccard index can be used to calculate the size of the intersection (i.e., overlapping elements) and the remaining union (i.e., non-overlapping elements) between the two databases. When combined with the known precision for IBD, the total IBD cohort size can be estimated (assuming that the precision is the same for both the intersection and union and that complex interactions do not exist within databases). This method is mathematically and clinically useful only for calculating an upper estimate when more than two databases are analyzed. With this caveat in mind, the inference protocol is described in the next section.

#### Cohort Size Inference Protocol

1. Start with the primary database (ICD10 codes) and multiply the unique patients in this dataset by the precision of this dataset to obtain a base ‘Combined’ predicted IBD set.
2. Sort other databases by precision (descending).
3. Iterate over these databases in order as follows:
  - Pick the following highest-precision dataset that has not yet been integrated.
  - Calculate the Jaccard index between the current ‘Combined’ and the next highest-precision set.
  - Use the recalculated Jaccard similarity index between ‘Combined’ and the following dataset to estimate the unique patients contributed by that dataset (i.e., those only present on the new dataset’s side of the union).
  - The unique patients in that dataset are multiplied only by the dataset’s precision to estimate the incremental true positives.
  - Add that unique set of patients to the ‘Combined’ set.
  - Repeat the process until no more datasets remain.

Although this process may seem elegant, it has significant weaknesses. Primarily, it assumes that precision, assessed against the gold standard, is equally weighted between patients at the intersection and those only in the union. This assumption leads to the method overestimating the total cohort size. The code for this algorithm is provided as fully open source at this URL to maximize transparency and replicability.

### Multivariate Modeling

Machine learning (ML) logistic regression (LR) [36] classifiers were constructed using 11 available databases. Demographic features such as age, sex, ethnicity, and IMD decile were excluded from the feature set using predefined patterns. Features were standardized using z-scores (mean = 0, standard deviation = 1) before model fitting. To improve the algorithm’s performance, the L2 (ridge) penalty was used in conjunction with the regularized least absolute shrinkage and selection operator (L1) (lasso) penalty in a 50:50 elastic net mix to evaluate the features that could enhance the prediction. The lasso shrinks parameters according to their variance, reducing overfitting and enabling automatic variable selection [39]. At the same time, the ridge L2 penalization provides stability as it does not allow any feature coefficient to shrink to zero. The optimal degree of regularization was determined by identifying a tuning parameter λ using nested cross-validation (as described below) with a stochastic average gradient augmented (SAGA) solver in light of the sparsity of the underlying data (primarily due to negative imputation). To avoid overfitting and reduce the number of false-positive predictors, λ was selected to provide a model with an area under the receiver operating characteristic curve (AUC) and one standard error below that of the best model.

All analyses used pandas, fairlearn, numpy, seaborn, matplotlib, and scikit-learn packages in VS Code™ and Python 3.10.10 with poetry to manage virtual environments. The code was version-controlled using Git and made available open-source to maximize replicability and transparency.

#### Cross-Validation and Calibration

To evaluate the model’s predictive performance for new cases of the same underlying population (internal validation), nested cross-validation (tenfold for the inner loop; tenfold/100 repeats for the outer loop) was performed. Platt scaling was used to improve the calibration [37] because the calibration distribution was approximately sigmoid in shape. Discrimination was assessed using the AUC and Brier scores [38]. All steps (feature selection, scaling, and threshold selection) were incorporated into the model development and selection process to avoid data leakage, which would otherwise result in optimistic performance measures. Type 2a validation was performed on the holdout set [39].

Measures of discrimination (precision, recall, harmonic F1 score, Brier score) and calibration were assessed. Calibration was evaluated using three methods.

1. A standard calibration curve plotting mean predicted probabilities against observed proportions in bins.
2. A locally estimated scatterplot smoothing (LOESS) calibration curve was fitted to the predicted probabilities and observed outcomes.
3. A logistic regression calibration plot fitting a logistic regression curve to the same data.

### Bias Identification/Error Analysis

The model’s potential for bias was also examined through a stratified analysis of its performance across different demographic groups (race, age, sex, and Index of Multiple Deprivation (IMD)) and by comparing the AUC for these subgroups.

## Results

### Total Study Cohort

Between 2007 and 2023, 52,332 non-two-week wait referrals were made for 37,947 individual patients. The gold-standard validation cohort comprised 2800 patients: 2092(75%) with IBD and 708(25%) non-IBD cases. The randomly seeded validation subset (30%) consisted of 840 patients, 628 (74.8%) with IBD and 213 (25.4%) without IBD.

**Fig. 1:**
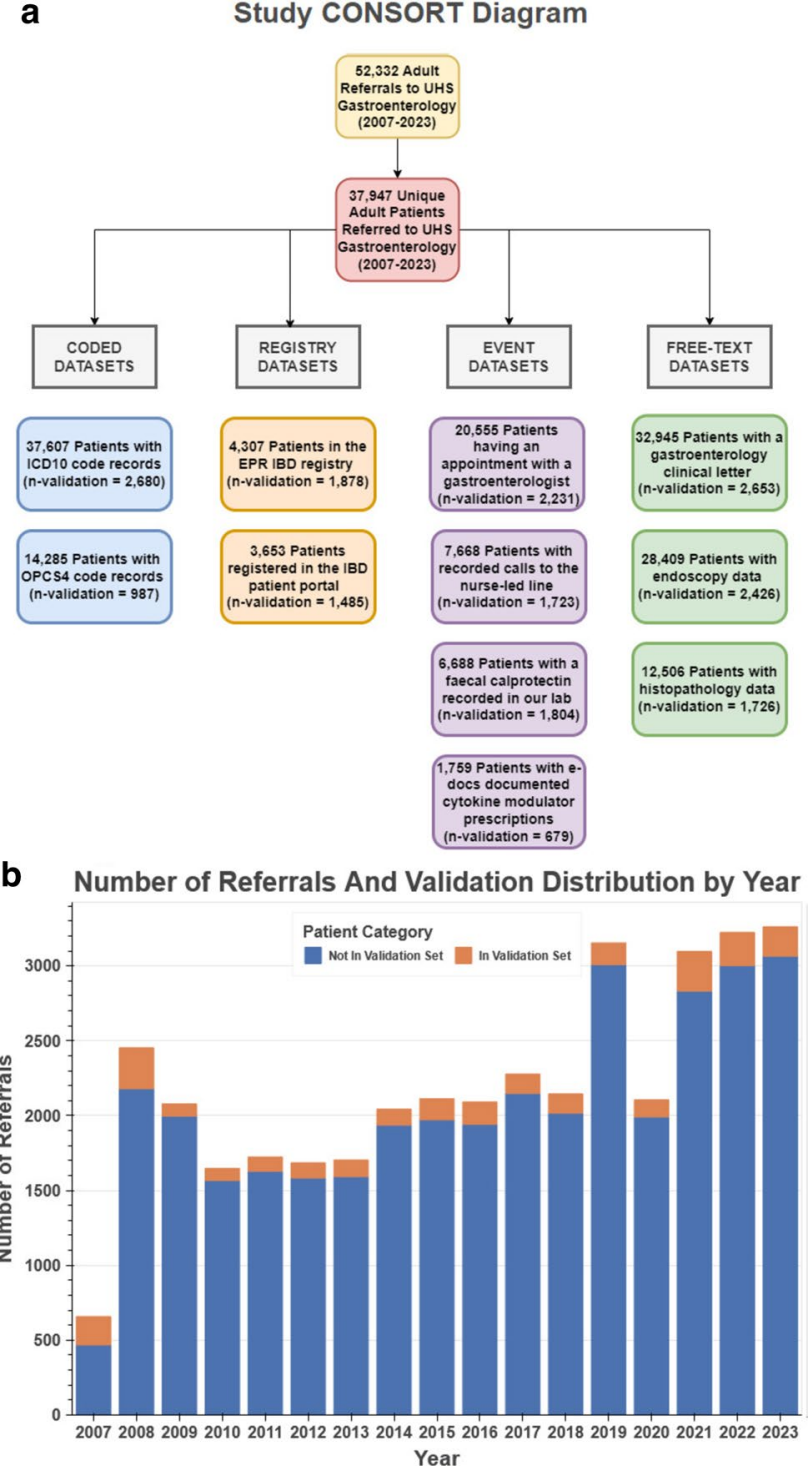
Study population CONSORT and temporal distribution. **a** Describes the distribution of patients in the study by database and database type. **b** Illustrates the temporal distribution of patients by year of first referral to Gastroenterology, showcasing the validation cohort’s distribution throughout the study period, with some notable fluctuations, particularly in 2019, 2020, and the initial 2 years of the PAS’s implementation at the trust (2007–2008)

Figure 1 shows the distribution of these patients in each database and temporally by year of the first referral to the gastrointestinal service.

#### Cohort Demographics

Table 2 shows the demographic characteristics of the entire cohort. The skewness and kurtosis for age were 0.017 and − 1.076, respectively, and those for IMD were − 0.111 and − 1.181, respectively.

**Table 2:** Full cohort demographics

Feature	Mean	Median
Age at point of referral	51.79yrs (95%CI 51.59–51.92)	52.22yrs (IQR: 32.4)
Sex (female)	60.27%	
Ethnicity (white)	85.04%	
IMD decile	5.91 (95%CI 5.88–5.94)	6 (IQR: 4)
Urgent referrals	21.34%	
Local referrals From Southampton Catchment	83.01%	

Describes the cohort demographic characteristics of the patients included in the study

### Coding, Event and Registry-Based Predictions

By examining the relationships between each dataset and the gold-standard validation cohort, the baseline precision and recall were established for each database, as shown in Table 3.

**Table 3:** Coding, registry, and event-based predictions

Database	Coverage	Accuracy	Precision	Recall	Specificity	NPV	F1 Score
Coding							
ICD10 Codes	802 (95.48%)	0.93 (0.91–0.95)	**0.96 (0.94**–**0.98)**	0.95 (0.92–0.97)	0.86 (0.81–0.91)	0.85 (0.80–0.90)	0.96 (0.94–0.97)
OPCS4 Codes	274 (32.6%)	0.33 (0.28–0.38)	**0.86 (0.76**–**0.96)**	0.17 (0.12–0.22)	0.90 (0.82–0.98)	0.23 (0.18–0.28)	0.29 (0.22–0.35)
Registries							
Patient Portal	428 (50.95%)	0.97 (0.95–0.99)	**0.97 (0.95–0.99)**	1.0 (1.0–1.0)	0.0 (0.0–0.0)	0 (0.0–0.0)	0.98 (0.97–0.99)
EPR IBD Registry	571 (67.98%)	0.97 (0.96–0.98)	**0.97 (0.96**–**0.98)**	1.0 (1.0–1.0)	0.0 (0.0–0.0)	0 (0.0–0.0)	0.99 (0.98–1.0)
Event							
Cytokine Modulator Prescriptions	198 (23.57%)	1.0 (1.0–1.0)	**1.0 (1.0**–**1.0)**	1.0 (1.0–1.0)	1.0 (1.0–1.0)	0.0 (0.0–0.0)	1.0 (1.0–1.0)
Flare Calls	505 (60.12%)	0.87 (0.84–0.90)	**0.87 (0.84**–**0.91)**	1.0 (1.0–1.0)	1.0 (1.0–1.0)	0 (0.0–0.0)	0.93 (0.91–0.95)
IBD Clinic Appointments	664 (79.05%)	0.63 (0.59–0.67)	**0.80 (0.76**–**0.86)**	0.70 (0.66–0.74)	0.34 (0.26–0.42)	0.23 (0.18–0.28)	0.75 (0.71–0.77)
Calprotectin > 50	533 (63.45%)	0.63 (0.0.59–0.67)	**0.80 (0.76**–**0.84)**	0.70 (0.66–0.74)	0.34 (0.26–0.42)	0.23 (0.18–0.28)	0.75 (0.72–0.78)

Baseline ground truth established using the validation cohort, which compares coverage, precision, and recall for each dataset. The F1 score is provided for each dataset

### Simple String Regex Search Model

The string regression search model is the most straightforward natural language processing (NLP)-based cohort identification model. It was used as a proxy for the likelihood of IBD among free-text documents, and Table 4 displays the results below.

**Table 4:** String search model comparison

Database	Coverage	Accuracy	Precision	Recall	Specificity	NPV	F1 score
Endoscopy records	738 (87.9%)	0.73 (0.70–0.76)	**0.95 (0.93**–**0.97)**	0.70 (0.66–0.74)	0.86 (0.81–0.91)	0.41 (0.35–0.47)	0.80 (0.77–0.83)
Clinical letters	794 (94.5%)	0.80 (0.77–0.83)	**0.79 (0.76**–**0.82)**	0.99 (0.98–1.0)	0.14(0.09–0.19)	0.96 (0.89–1.0)	0.88 (0.86–0.90)
Histopathology records	506 (60.24%)	0.68 (0.70–0.72)	**0.73 (0.69**–**0.77)**	0.89 (0.86–0.92)	0.15 (0.09–0.21)	0.35 (0.24–0.46)	0.80 (0.77–0.83)

Describes the results of the regex string search models across clinical, endoscopy, and histopathology records.

### Database Cardinality

**Fig. 2:**
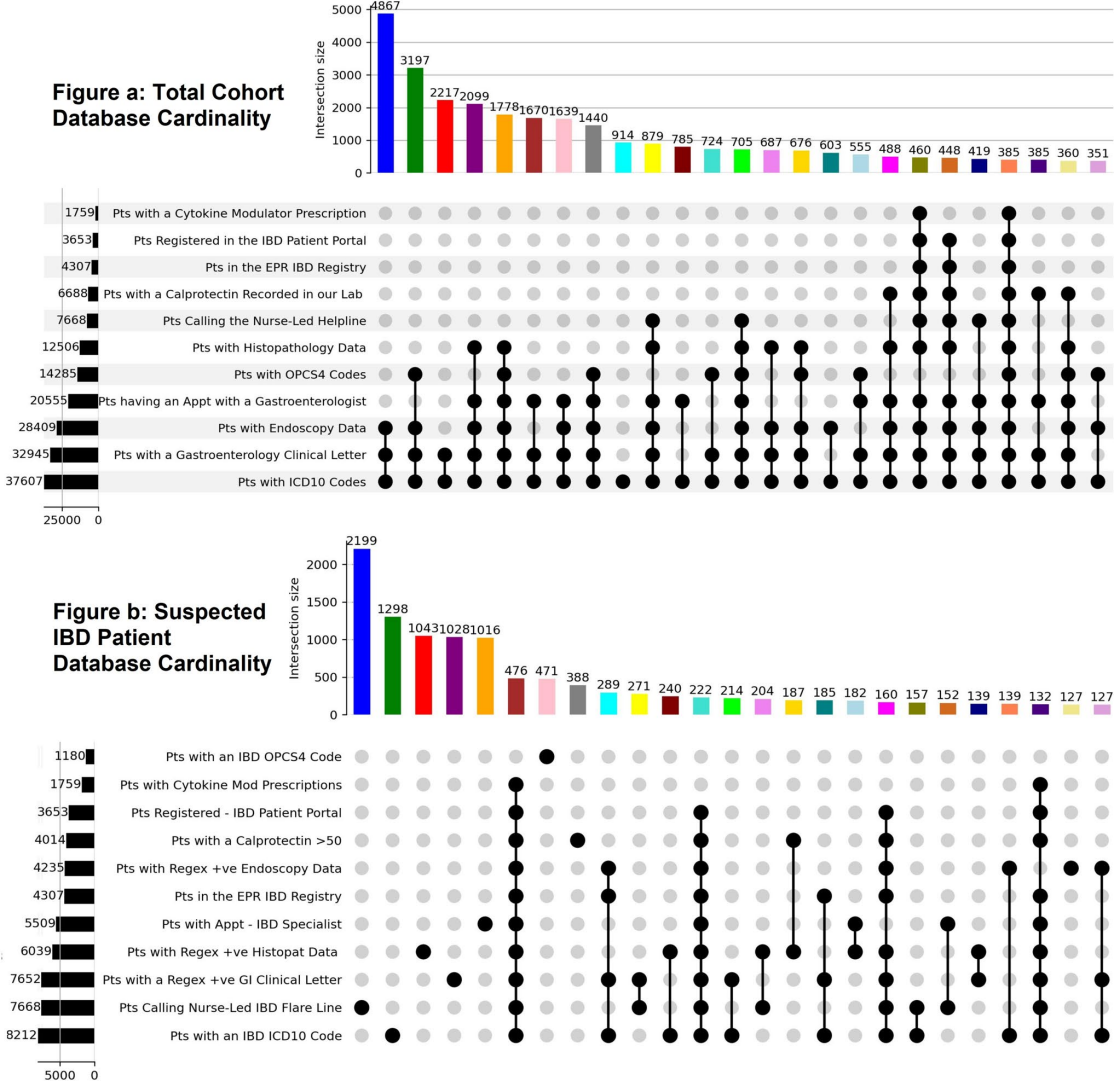
Database cardinality comparison (UpSet plots). Two UpSet plots are displayed above. Figure **a** describes database cardinality among the total cohort, while Figure **b** illustrates database cardinality among the patients suspected of having IBD. Both figures show high overall database cardinality, with only 385 (1%) of patients having records in all 11 databases.

Cardinality measures the uniqueness or distinctiveness of elements within a database table. Given this study’s sheer number of intersecting sets, the results were best visualized using UpSet plots (Fig. 2). These are superior to Venn diagrams for visualizing datasets with more than three intersecting sets in a matrix.

Significant overlaps between suspected IBD cases in databases are the exception rather than the rule here. Of the 8212 unique patients with at least one ICD-10 code for IBD, only 476 (5.8%) were found in ten or more clinical databases.

### Jaccard Similarity Indices

**Fig. 3:**
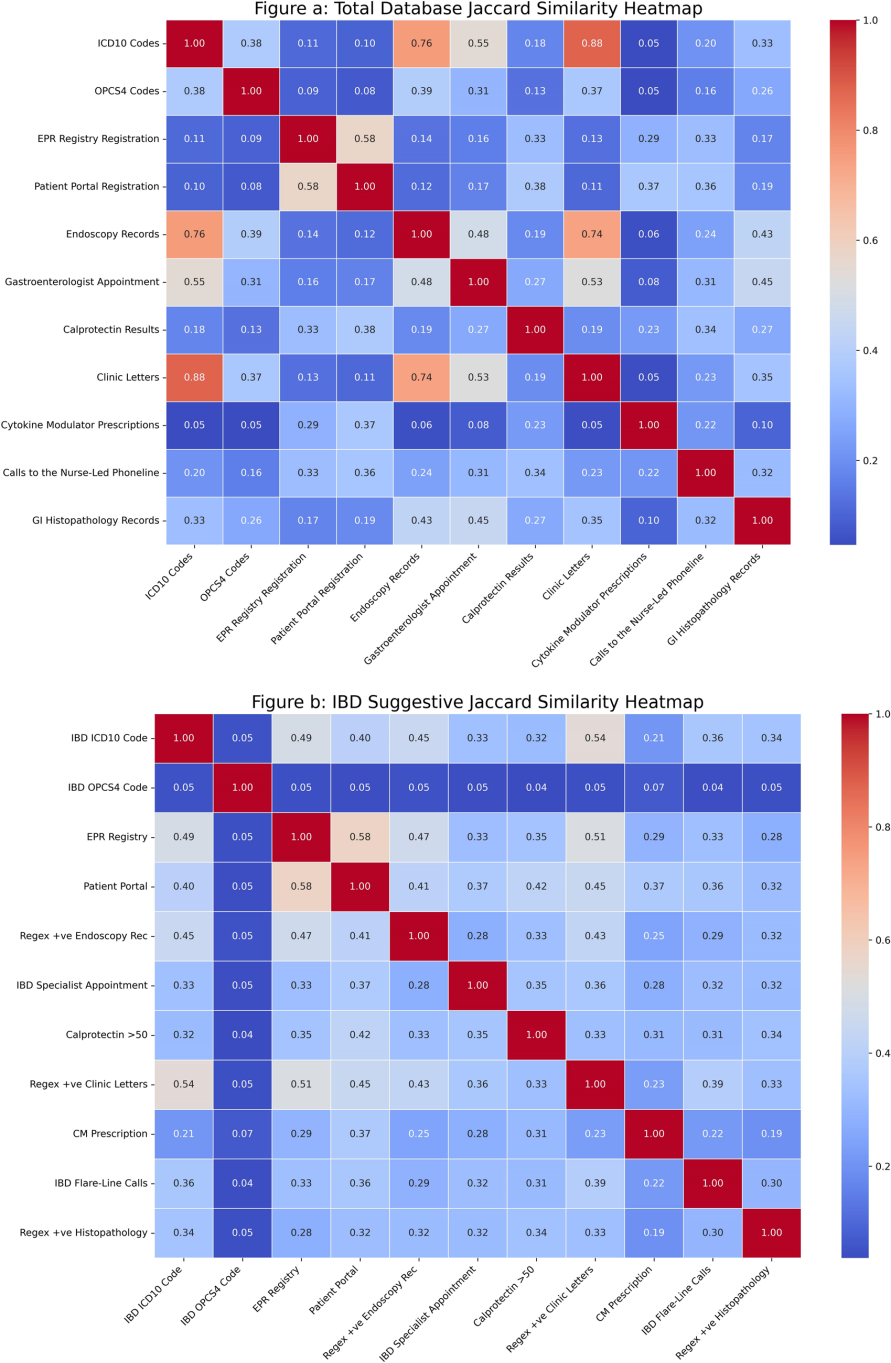
Heatmap of Jaccard similarity indices across databases. Highlights database correlations by Jaccard similarity indices across both the total datasets and for the patients who are more likely to have IBD

Figure 3 shows the Jaccard indices [30] across all 11 databases before and after the application of IBD prediction criteria.

Jaccard indices were high for the complete clinical coding database, clinical letters (0.88), and endoscopy records (0.76). The same was true for endoscopy and clinical letters (0.74). Moderate to high indices existed between coding and appointments (0.55), OPCS4 codes and ICD10 coding (0.38), endoscopy (0.39), and clinic letters (0.37). Moderate correlations existed between patient portal registration and registration within EPR (0.58), flare calls (0.36), calprotectin testing (0.37), and cytokine modulator usage (0.38). The same was observed between endoscopies and appointments (0.48) as well as between GI histopathology (0.43) and appointments. In comparison, appointments intersected with clinical letters (0.53) and histopathology (0.45).

However, these indices were substantially altered in the suspected IBD cohort, with no high Jaccard indices. IBD ICD10 codes intersected moderately with the registry databases (0.4–0.49), clinic letters (0.54), and endoscopies (0.45). The EPR registry intersects with endoscopy (0.47) and clinical letters (0.51), whereas the patient portal intersects with flare line calls (0.38), cytokine modulator prescriptions (0.37), clinical letters (0.45), calprotectins > 50 (0.42), IBD specialist appointments (0.37), and endoscopy (0.41). Endoscopy overlapped with clinical letters suggesting IBD (0.43). IBD specialist appointments also overlapped with clinical letters suggestive of IBD (0.36). The remainder of the database intersections are low, ranging (0.19–0.35), except for IBD OPCS4 codes, where the range is even lower (0.04–0.07).

### Cohort Size Estimation by Recursive Jaccard Similarity Database Inference

The full results of the inference process are described in Table 5*.*

**Table 5:** Full recursive Jaccard similarity cohort size estimation

Database	Flagged Positive Cases	Jaccard with Combined	Intersection	Unique	Precision	Incremental TPs	Cumulative TPs
ICD10 Codes	8337			8337	0.96	8004	8004
Cytokine Modulator Prescriptions	1762	0.205	1718	44	1.00	44	8048
Patient Portal	3643	0.408	3483	160	0.97	155	8203
EPR IBD Registry	4312	0.501	4288	24	0.97	23	8226
Endoscopy Records	4327	0.447	3982	345	0.95	328	8554
Flare Calls	7705	0.363	4428	3277	0.87	2851	11,405
OPCS4 Codes	1190	0.046	592	598	0.86	514	11,919
IBD Clinic Appointments	5520	0.282	4021	1499	0.80	1199	13,118
Calprotectin > 50	4000	0.228	3398	602	0.80	482	13,600
Clinical Letters	14,984	0.485	9757	5227	0.79	4129	17,729
Histopathology Records	6070	0.257	5352	718	0.73	524	18,253
All Integrated	20,831						**18,253**

Describes the results of the recursive cohort size estimation strategy pursued according to the defined protocol

*TP’s* true positives

The largest unaccounted-for group emerged from clinic letters (*n* = 4129), followed by flare calls (*n* = 2851). These factors alone accounted for 6980 additional patients with uncoded IBD.

### Cohort Size Estimation by Penalized Logistic Regression

The following estimates of the total IBD population size, as shown in Table 6, were obtained by applying thresholding to the penalized logistic regression (LR) model.

**Table 6:** IBD logistic regression (LR) predictions by threshold

Threshold	Precision (95%CI)	Recall (95%CI)	Accuracy (95%CI)	Predicted IBD total (95%CI)	Actual predicted IBD total (95%CI)
0.25	0.8 (0.78–0.81)	0.92 (0.91–0.93)	0.76 (0.75–0.78)	18,590 (18,416–18,778)	14,872 (14,531–15,190)
0.31	0.8 (0.79–0.82)	0.92 (0.91–0.93)	0.77 (0.75–0.78)	18,511 (18,321–18,700)	14,809 (14,484–15,142)
0.38	0.83 (0.81–0.84)	0.92 (0.91–0.93)	0.79 (0.78–0.81)	11,658 (11,487–11,821)	9676 (9415–9866)
0.44	0.83 (0.82–0.85)	0.92 (0.91–0.93)	0.8 (0.78–0.81)	9880 (9720–10,047)	8200 (8000–8414)
0.5	0.84 (0.82, 0.85]	0.92 (0.9–0.93)	0.8 (0.78–0.81)	9487 (9318–9645)	7969 (7724–8136)
0.56	0.83 (0.82–0.85)	0.91 (0.89–0.92)	0.79 (0.78–0.81)	9433 (9251–9594)	7829 (7679–8070)
0.62	0.85 (0.83–0.86)	0.9 (0.89–0.92)	0.8 (0.79–0.82)	9308 (9132–9466)	7912 (7677–8068)
0.69	0.86 (0.85–0.87)	0.9 (0.89–0.91)	0.81 (0.8–0.83)	8999 (8847–9155)	7739 (7551–7947)
0.75	0.87 (0.86–0.88)	0.89 (0.88–0.91)	0.82 (0.8–0.83)	8692 (8533–8857)	7562 (7390–7767)

Describes the results of thresholding the LR model at different levels

The optimal threshold was 0.4964, detecting 8,159 true-positive patients with IBD with a global AUROC of 0.85 against the validation set. Adding only the unaccounted-for true positives from a single database (clinic letters) to this total (*n* = 4889) resulted in a final estimated total of **13,048** true-positive patients with IBD.

### Final Model Coefficients

It is not possible to be fully transparent about performance without also examining the LR model coefficients and corresponding odds ratios, as highlighted in Table 7.

**Table 7:** Final Model Coefficients

Feature	Co-efficient	Odds ratio
Intercept	2.232	
IBD suggestive ICD-10 diagnosis codes	0.958	2.607
IBD suggestive electronic patient record (EPR) IBD Registry	0.837	2.310
IBD suggestive IBD patient portal	0.782	2.187
IBD -suggestive cytokine modulator prescriptions	0.511	1.666
IBD suggestive endoscopy reports	0.165	1.179
IBD suggestive flare line calls	0.027	1.027
IBD suggestive OPCS-4 surgical procedure codes	0	1
IBD suggestive clinic appointments	− 0.033	0.967
IBD suggestive fecal calprotectins	− 0.048	0.953
IBD suggestive gastrointestinal histopathology	− 0.292	0.747
IBD suggestive gastroenterology clinic letters	− 0.427	0.653

Above explores the ranked coefficients and odds ratios within the final LR model

The model prioritizes ICD10 codes and registry/drug data to make its predictions. Flare calls, clinic appointments, and calprotectins have minimal predictive weight, and OPCS4 code weights are zeroed. Unstructured data sources are mainly weighted negatively in the model (except for endoscopy), resulting in underprediction for these groups. These results suggest that the approach taken here is both explainable and robust because removing microscopic colitis patients and re-running the experiment did not break the model.

#### Calibration and Bias

**Fig. 4:**
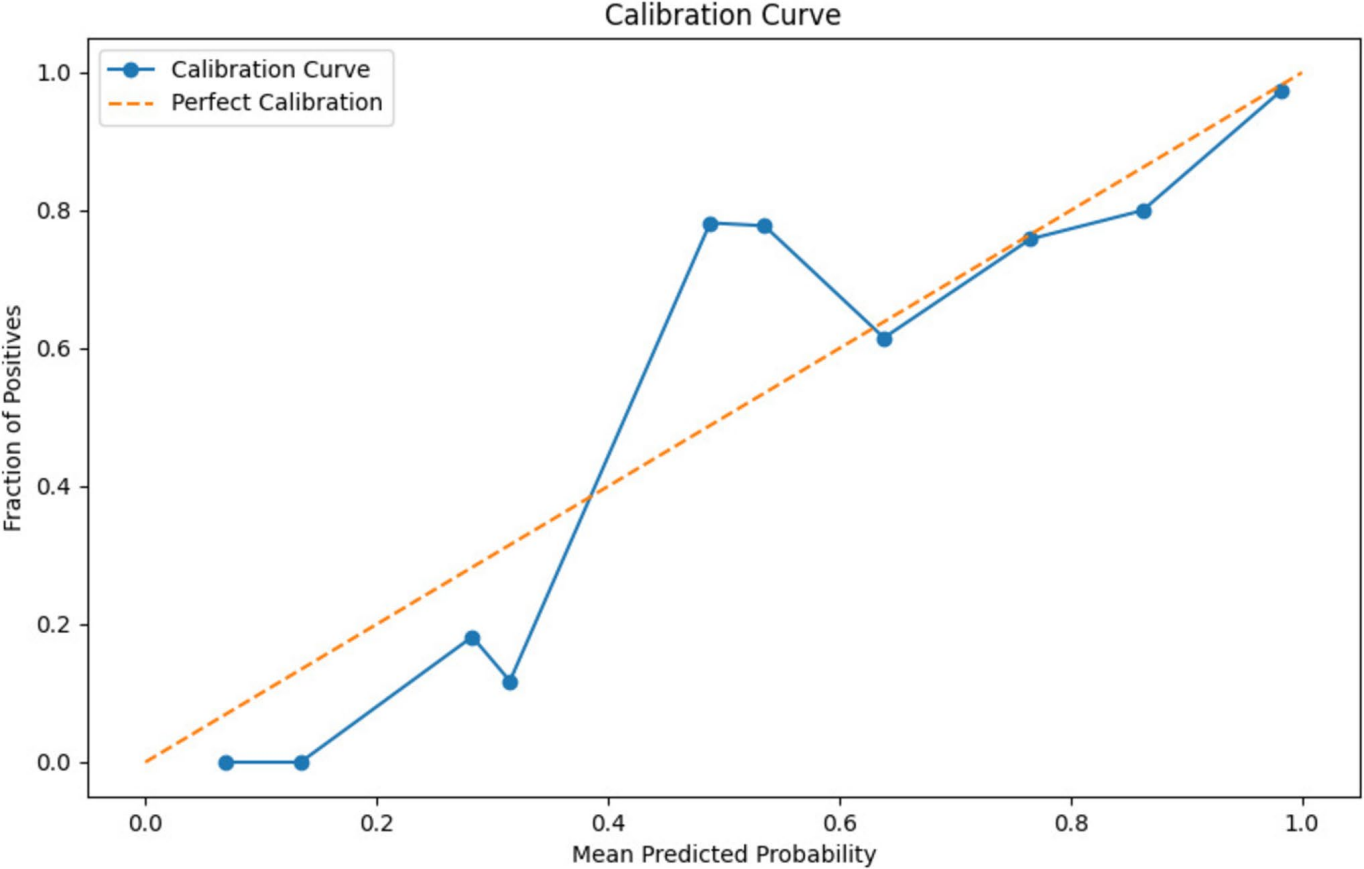
Model calibration curve. Demonstrates the calibration curve for the LR model. With the existing database covariates, it is not feasible to calibrate the model perfectly

Calibration remains an issue for the model despite retraining. Figure 4 demonstrates that the model still tends to underpredict patients with IBD at lower predicted probabilities but overpredicts above 0.4, despite Platt scaling, which initially improved the Brier score from 0.0515 to 0.0461. However, removing the microscopic colitis patients from the IBD cohort in the second iteration, following peer review, then increased the Brier score back to 0.0620 by increasing the difficulty of the prediction challenge.

No significant bias was detected in this model for levels of deprivation or sex. However, the model performed significantly better among Caucasians (AUC-0.87) than among Asians (AUC-0.81) or Africans (AUC-0.76). Additionally, performance decreased in older age groups, declining from 0.90in the 20–39 age range to 0.83 for those aged 50–59.

## Discussion

Accurate cohort data have substantial implications for policy formation, departmental resource allocation, and the avoidance of discrimination in patient care, research, and service improvement. This study highlights significant flaws in relying solely on billing/read codes and medication data within secondary care to identify clinical IBD cohorts at local, regional, and population levels. Only 8,048 (61.7%) patients were identifiable from a combination of billing codes and medication data from an actual local IBD patient cohort of likely up to *13,048* individuals.

The major strengths of this work include its real-world nature, a robust approach to validation, the variety of databases investigated, and the simplicity of the methodology, which facilitates replicability in other settings without requiring advanced data science capabilities. This study has revealed significant flaws in the current assumptions underlying the identification of IBD clinical cohorts. Further cohort capture can be achieved by adding additional databases if sensible defragmentation attempts are made.

Weaknesses include the study’s single-center nature, which means that the prevalence cannot be accurately calculated, and the relative weakness of the NLP methods employed (simple regular expressions), which may have led to the inclusion of other forms of colitis in the cohort. This problem will be addressed in the follow-up study. Actual positive cases are unlikely to be equally distributed between intersections and unions because the patients in the validation cohort were randomly drawn from a higher-probability IBD sample (present in at least two databases at the outset). This means that they did not adequately represent the cohort fringes, where only a single database node value existed, leading to performance issues in low-prevalence settings (< 0.3), which caused the LR model to underpredict these cases. Using a different classifier type, such as a tree or neural network classifier, or another scaling method, cannot overcome this edge-node problem, which is inherent to the gold-standard cohort. Additionally, this single-edge-node problem cannot be overcome by adding additional features (such as age, ethnicity, and IMD) to the model. Such attempts typically compounded the calibration problem without substantially ameliorating model biases.

Because two-week wait referrals were excluded from this study, the cohort will likely be even larger than reported here. Additionally, clinical information (e.g., clinical letters scanned as images) was unavailable for analysis in this study, suggesting that even more patients may be retrievable had optical character recognition been in place.

Clinical letters are the most critical contributor to missing patients outside the LR model trained here. Montoto et al. [39] (2022) claimed to achieve 0.88 precision and 0.98 recall for diagnosing Crohn’s disease within a large Spanish multicenter cohort. However, the free-text precision of the simple regex algorithm we derived here was only 0.79, and the recall was 0.99 for detecting positive IBD cases across clinic letters. The comparatively lower precision is due to the straightforward approach taken here. However, in the Montoto study, even though they used a more sophisticated algorithm, their validation was underpowered, with only 100 records validated at each site and multiple variables predicted. They also did not use blinding or provide named grades for those performing the validation. It is also not clear exactly how they conducted their sampling. The algorithm derived here is, by contrast, completely explainable. Although the sampling was flawed in that it drew from a higher prevalence population of IBD (leading the model to underpredict lower prevalence cases), it was randomly distributed temporally across multiple databases of sufficient scale and fully transparently reported and documented.

This is the first time that the full severity/extent of the impacts of database fragmentation has been documented for IBD cohorts, building on the work of others [21, 22]. The principles of (1) exposing many different databases, (2) validating a gold-standard cohort, and (3) using ML to identify a complete cohort are transferable to most other clinical domains and diseases. However, to make this process more scalable in the future, novel methodologies are required to standardize datasets, positively identify patients, and compare databases across a graph of tables. Success in these endeavors will positively impact clinical research, population health, and frontline clinical care by highlighting the true IBD clinical cohorts of local teams.

## Conclusion

Diagnostic billing codes and medication data alone cannot accurately identify complete cohorts of individuals with IBD in secondary care. A multimodal cross-database model can partially compensate for this deficit. However, it cannot capitalize on the clinic letters without more robust natural language processing (NLP)-based identification mechanisms being in place. Future work will focus on solving this problem.

## Supplementary Information

Below is the link to the electronic supplementary material.

Supplementary file1 (PDF 39 KB)

Supplementary file2 (PDF 88 KB)

## Data Availability

All data generated or analyzed during this study are unavailable to protect patient privacy as the patients involved did not consent to sharing their data.

## Code Availability

All codes used in the analytics for this project are made available open source on GitHub at https://github.com/MattStammers/IBD_Cohort_Size_Estimation_IC-IBD_Study_Part_1

## Declarations

### Model availability

The LR model developed in this project is available in the GitHub repo as a.pkl file. Some setup is required to be useful in other contexts, but it is made available on a CC-BY-NC 4.0 basis.

### Patient and public involvement

An IBD patient from the local patient panel helped develop the ethics application and study protocol.
