# Supplementary material for: Identification of Cohorts with Inflammatory Bowel Disease Amidst Fragmented Clinical Databases via Machine Learning
Source: Dig Dis Sci. 2025 Aug 13;70(10):3309–22. doi: 10.1007/s10620-025-09323-1 (PMC12531273; doi:10.1007/s10620-025-09323-1)
Supplement: Supplementary file 2 — Supplementary file2 (PDF 88 KB) [file 10620_2025_9323_MOESM2_ESM.pdf]

<u>Supplement 2 – OPCS4 Codes Relevant to IBD as per RCP Audit</u>

CHAPTER G - SMALL INTESTINE (CODES G58-G82)

- **Jejunum (G58-G67)**
- **G58 Excision of Jejunum**
    - G58.1 Total Jejunectomy and Anastomosis
    - G58.2-9 Other Specified and Unspecified
- **G59 Extirpation of Lesion of Jejunum**
    - G59.1 Excision of Lesion
    - G59.2-9 Other Specified and Unspecified
- **G60 Artificial Opening into Jejunum**
    - G60.1 Creation of Jejunostomy
    - G60.2-9 Other Specified and Unspecified
- **G63 Other Open Operations on Jejunum**
    - G63.1 Open Biopsy
    - G63.2-9 Other Specified and Unspecified
- **G67 Other Operations on Jejunum**
    - G67.1 Intubation for Decompression
    - G67.8-9 Other Specified and Unspecified
- **Ileum (G69-G82)**
- **G69 Excision of Ileum**
    - G69.1 Ileectomy and Anastomosis
    - G69.2-9 Other Specified and Unspecified
- **G70 Open Extirpation of Lesion of Ileum**
    - G70.2-9 Other Specified and Unspecified
- **G71 Bypass of Ileum**
    - G71.1-5 Anastomosis Variations
    - G71.8-9 Other Specified and Unspecified
- **G72 Other Connection of Ileum**
    - G72.1-5 Anastomosis Variations
    - G72.8-9 Other Specified and Unspecified
- **G73 Attention to Connection of Ileum**
    - G73.1-9 Revision, Closure, and Other Specified
- **G74 Creation of Artificial Opening into Ileum**

    - G74.1-9 Variations and Unspecified
- **G75 Attention to Artificial Opening into Ileum**
    - G75.1-9 Refashioning, Repair, and Other Specified
- **G78 Other Open Operations on Ileum**
    - G78.1-9 Biopsy, Strictureplasty, and Other Specified
- **G82 Other Operations on Ileum**
    - G82.2-9 Intubation for Decompression and Other Specified
- **CHAPTER H - LOWER DIGESTIVE TRACT (CODES H01-H62)**
- **Appendix (H01-H03)**
- **H01 Emergency Excision of Appendix**
    - H01.1-9 Various Types and Unspecified
- **H02 Other Excision of Appendix**
    - H02.1-9 Interval, Planned, and Other Specified
- **H03 Other Operations on Appendix**
    - H03.1-9 Drainage and Other Specified
- **Colon (H04-H30)**
- **H04 Total Excision of Colon and Rectum**
    - H04.1-9 Panproctocolectomy Variations and Unspecified
- **H05 Total Excision of Colon**
    - H05.1-9 Total Colectomy Variations and Unspecified
- **H06 Extended Excision of Right Hemicolon**
    - H06.1-9 Various Anastomosis and Ileostomy
- **H07 Other Excision of Right Hemicolon**
    - H07.1-9 Hemicolectomy Variations
- **H08 Excision of Transverse Colon**
    - H08.1-9 Colectomy and Anastomosis Variations
- **H09 Excision of Left Hemicolon**
    - H09.1-9 Hemicolectomy and Anastomosis Variations
- **H10 Excision of Sigmoid Colon**
    - H10.1-9 Colectomy and Anastomosis Variations
- **H11 Other Excision of Colon**
    - H11.1-9 Colectomy and Anastomosis Variations
- **H12 Extirpation of Lesion of Colon**
    - H12.2-9 Excision, Destruction, and Other Specified

- **H13 Bypass of Colon**
  - H13.1-9 Anastomosis Variations and Unspecified
- **H14 Exteriorisation of Caecum**
  - H14.1-9 Caecostomy Variations and Unspecified
- **H15 Other Exteriorisation of Colon**
  - H15.1-9 Colostomy Variations and Unspecified
- **H16 Incision of Colon**
  - H16.1-9 Drainage, Colotomy, and Other Specified
- **H19 Other Open Operations on Colon**
  - H19.1-9 Biopsy and Other Specified
- **H30 Other Operations on Colon**
  - H30.8-9 Other Specified and Unspecified
- **Rectum (H33-H46)**
- **H33 Excision of Rectum**
  - H33.1-9 Various Excisions and Anastomosis
- **H34 Open Extirpation of Lesion of Rectum**
  - H34.1-9 Various Types and Unspecified
- **H40 Operations on Rectum through Anal Sphincter**
  - H40.1-9 Various Types and Unspecified
- **H41 Other Operations on Rectum through Anus**
  - (Specific codes not provided)
- **H46 Other Operations on Rectum**
  - (Specific codes not provided)
- **Anus and Perianal Region**
- **H47 Excision of Anus**
- **H48 Excision of Lesion of Anus**
- **H49 Destruction of Lesion of Anus**
- **H54 Dilation of Anal Sphincter**
- **H55 Other Operations on Perianal Region**
- **H56 Other Operations on Anus**
